# Peer Feedback in Translation Training: A Quasi-Experiment in an Advanced Chinese–English Translation Course

**DOI:** 10.3389/fpsyg.2021.631898

**Published:** 2021-07-28

**Authors:** Zhong Lin, Xinyu Song, Jingwen Guo, Feng Wang

**Affiliations:** ^1^School of Foreign Language Studies, Chang'an University, Xi'an, China; ^2^School of Education, University of Bristol, Bristol, United Kingdom; ^3^School of Foreign Languages, Xidian University, Xi'an, China

**Keywords:** peer review, peer feedback, translation teaching, second language learners, Chinese–English translation

## Abstract

Although research on peer feedback in second language teaching and learning has been developed from various perspectives over the past three decades, less is known about feedback in translation settings. This study reports the results of a quasi-experiment with advanced second language learners in a Chinese–English translation course. It examines how effective peer feedback is in improving the quality of translations. The following data were collected from 30 students: their initial translation drafts, the drafts with the feedback of their peers, and the final corrected translations. The whole process was facilitated by computer assistance and under anonymity. It was found that most students drew on direct or indirect corrective feedback while few students drew on metalinguistic corrective feedback. Text genres were also proved to impact the types and counts of peer feedback. An analysis of the accuracy rate of corrections after peer feedback showed that it had a positive impact on translation quality. The findings shed light on the applicability of peer feedback in other pedagogical activities.

## Introduction

Peer feedback is an essential activity in L2 teaching and learning and has gained much attention since the 1990s (Nelson and Murphy, 1992; Connor and Asenavage, 1994). Since teachers find it time-consuming to correct the assignments of each student (Shen et al., 2017), organizing peer feedback can ease their tutoring burden. An additional benefit is that it helps students from a social, linguistic, cognitive, or affective perspective (Cao et al., 2019).

However, in-depth research has scarcely been done on peer feedback effects on translation teaching and learning (Min, 2006). Most of the studies relate to writing courses, and there is still a gap regarding its role in other skills. Minimal knowledge is available on the process and impact of feedback on translation settings. This study explores peer feedback in an advanced Chinese–English translation class to investigate how peer feedback contributes to translation achievement.

## Literature Review

### Peer Feedback

Peer feedback varies in scope, content, and style (cf. van den Bos and Tan, 2019). Researchers have categorized peer feedback into three levels. The first refers to implementable (or corrective) feedback that focuses on task performance. This type of feedback identifies specific errors. The second centers on understanding and learning of texts and is called information processing feedback. Typical examples of this level include general identification of problems, general suggestions, and solutions. The third goes to personal evaluation, in which feedback takes the form of mere compliments and has nothing to do with implementing corrections. This level was considered to be the most ineffective by Hattie and Timperley (2007).

Ellis (2009:78) distinguished the feedback of teachers from that of the peer students. For the latter, he subdivided different forms of written corrective feedback, which by explicitness falls into three categories, namely, direct feedback, indirect feedback, and metalinguistic feedback. Direct corrective feedback stands for deleting redundant parts and replacing inappropriate words or phrases, while indirect and metalinguistic feedback offers no specific correction options. Indirect feedback may include underlining or drawing a circle to indicate an error, as well as the use of “modification symbols” to indicate omissions. Unlike indirect feedback, metalinguistic feedback shows the error using a code (e.g., “art” for “article”) or a short grammatical description.

Reversely, Ellis coded corrective feedback into “focused” or “unfocused,” depending on whether all types of errors or only specific ones were corrected. Another pair of the category of corrective feedback is electronic feedback and reformulation. The former refers to feedback that contains information that software programs can access, and the latter refers to markers that suggest reformulating the entire text.

### Peer Feedback in L2 Teaching and Learning

Peer feedback has been viewed as an acknowledged language pedagogy activity (Wang and Han, 2013). Since the 1990s, researchers have investigated the role of peer feedback in L2 teaching and learning, advocating the effectiveness of it from a social, linguistic, cognitive, or affective perspective. Several researchers listed specific benefits by comparing peer feedback with self-correction and teacher feedback.

The study of Connor and Asenavage (1994) of the influence of English as a second Language (ESL) writing groups found that the revision proportion by students from peer feedback was relatively small compared with those from teacher feedback and self-correction. They also found student reliance on teacher-centered classrooms could explain the low adoption rate of peer feedback because students might have a dubious attitude toward peer feedback, conceiving the comments of their peers would be wrong or that they would misinterpret their intentions (cf. Noroozi et al., 2016).

Despite the unwanted peer feedback, Zhang (1995) listed out four practical advantages of peer feedback over teacher comments as follows: it is more instructive, students gain more audience, learning attitudes of students improve, and it is effective in enabling them to make progress by evaluating the work of others. Yang (2010) also envisioned that differences between self-adjustments and peer comments were beneficial for students “…to monitor, evaluate, and adjust” when they wrote with the aim of achieving text improvement. Furthermore, peer feedback helped ease the anxiety of students and boost their confidence (Wang and Han, 2013).

Further research by Shen et al. (2017) substantiated the advantages of peer feedback as follows: first, peer feedback offered constructive suggestions, helping cognitive development of students; second, it improved language skills of students, both in terms of vocabulary and grammar; third, peer feedback seemed to promote meta-cognitive development of students by requiring them to think independently and critically; and finally, interacting with others helped develop their social skills. Researchers further argued that peer feedback contributed to the psychological development of students in which it could reduce the dependence of students on teachers and build up their self-confidence (Shen et al., 2020). At any rate, peer feedback is better than teacher correction in terms of “…timeliness, convenience, volume, and learner autonomy” (Wu and Schunn, 2020:2).

### Peer Feedback in Translation Classes

As discussed above, peer feedback applied to language writing has dominated the topic. In L2 writing, students typically compose on a specific topic, but their writings radically differ, where subjectivity plays a significant part. Diametrically, students in translation classes are given the same text to translate. They consequently review the assignments of their peers which are “…generally resemble their owns” (Wang and Han, 2013). Thus, the findings in writing peer feedback cannot be completely transferred to the translation context.

Lindgren et al. (2009) and Wang and Han (2013) investigated written translation peer feedback from the perspective of peer collaboration and concluded that peer feedback contributed to learner reflection of translation work. (Wang and Han, 2013) also discussed the perception of translation learners of their role in computer-aided peer feedback. The most recent study by Yu et al. (2020) investigated the written corrective feedback by teachers on English–Chinese translations based on the framework of Ellis (2009).

However, less is known about adopting peer feedback in advanced translation teaching (Wang and Han, 2013; Wu and Schunn, 2020). Wang and Han (2013) also claimed that more research should be done to assess the effectiveness of peer feedback in the translation performance improvement of students. Moreover, no study has discussed translation peer feedback from different source texts.

In conclusion, against the above background, this study aims to fill in the gap of a lack of study in peer feedback with computer assistance in an advanced Chinese–English translation class. The experiment of this study inquires the effect of different types of feedback with an emphasis on corrective feedback, where students are assumed to play two roles in peer feedback, i.e., receiver and giver (Cao et al., 2019).

## This Study

This study aims to address the following three research questions:

(1) What types of feedback do students use to correct the translation of others with computer assistance?
(2) To what extent does peer feedback, primarily corrective peer feedback, influence the final revisions of students?
(3) Do genres of translation texts impact peer feedback?

### Context and Participants

The context matters in peer feedback because the process and circumstances in which the task takes place can significantly influence its effectiveness (Ann, 1992). This study used a case-study methodology, which seemed appropriate for studying processes and interactions (Yin, 1984). We selected 30 third-year English students at Chang'an University (China) through both convenience and purposive sampling.

According to a pilot study, their English proficiency was advanced. The subjects were all native speakers of Chinese and had taken the same courses for five semesters, making their learning experience similar. Since the core courses (i.e., Advanced English, Translation, and Writing) lasted an entire academic year, they had already taken the English–Chinese translation course for one semester, where training in conducting peer feedback had been administered. They had often received feedback from the instructor on their assignments and had acquired a basic understanding of how to correct them.

The whole process was conducted online. We used electronic devices throughout the quasi-experiment to facilitate feedback, corrections, and data collection by the students. All participants wrote their drafts, provided feedback to others, and revised their final work with the word processing system (WPS).

Before the activity started, participants were given instructions and an example of how to give feedback was delivered. Since Carson and Nelson (1996) indicated that affective factors might divert peer feedback from error correction for the concern of community cohesion, all the materials in the experiment were in anonymity.

### Peer Feedback Execution

The peer feedback underwent the following three phases: preparation, feedback giving, and revision making (cf. van den Bos and Tan, 2019). Each phase was executed within a given period.

First, all participants received an introductory explanation of the purpose of the quasi-experiment, the tasks they had to complete, and the requirements for every single step, so that they were familiar with the template to follow. They were encouraged with the use of specific and understandable responses, such as “there is a grammatical problem in this sentence,” “there is information missing here,” “incorrect word usage here.” Unclear feedback such as “good job” and “well done” was to be avoided. To motivate student participation, 20% of the semester score was allocated to corrections of the work of others.

Then, they moved on to the second phase, i.e., providing peer feedback. Through random selection by the instructor, each student was assigned a translation draft to correct. In addition to correcting grammatical errors, we instructed them to make corrections for different interpretations of the source texts, word choices, etc. Students could also justify their corrections. Besides corrective feedback, they had to provide global comments. Students also had to label the place where the errors occurred. They could write their feedback in the margin or below the translation. All feedback was to be written in English.

During the final phase, participants revised their translation assignments based on their peer feedback and submitted the final translations. Students had to carefully consider each feedback point in this process, which weighed another 20% of the overall score of the semester. To facilitate further analysis, we required students to mark red for their revisions, blue for the unchanged items, and black for the rest. At the end of the quasi-experiment, we analyzed initial versions of all students, comments from peer feedback, and final translated texts.

### Data Collection and Analysis

During the semester in which this study was conducted, participants attended a 16-week Chinese–English translation course twice a week, for 90 min each time. The three translation tasks in this quasi-experiment followed the learning objectives of the course and were used as extra practice after class. To evaluate the translation performance of students in the most diverse way possible, we designed assignments in three different genres, namely, classical Chinese literature, a story in ancient Chinese, and an excerpt from a government report.

The data for this study covered the first translation assignment, second assignment with peer feedback, and their final revised translated texts of participants. In the end, we collected 30 sets of assignments. Based on the taxonomy of Ellis (2009), we further classified corrective peer feedback into direct, indirect, or metalinguistic (i.e., error code and short grammatical description) feedback. We recorded the number of types of corrective feedback before counting the number of total corrections and the percentage of corrected, reversed, and unchanged revisions in the final assignments of students. In addition to the degree of explicitness of the feedback, we classified the peer feedback into unfocused Written Corrective Feedaback (WCF) (highlighting specific language errors) or focused WCF (correcting all errors). Regarding the WCF strategies of students, we classified their corrections into electronic feedback (i.e., corrections available through software programs) or rewording (i.e., rewriting the entire text).

## Results

### Types of Peer Feedback in Chinese–English Translation Classes

The quasi-experiment yielded 512 feedback points. Of these, 70 (13.67%) fell into the personal evaluation category, 65 (12.70%) into the information processing category, and 377 (73.63%) into the corrective feedback category (see Table 1). Most students provided general comments about the translations that included both personal evaluation (i.e., compliments) and information processing feedback (i.e., identifying problems and providing general suggestions to improve the translation).

**Table 1:** Three levels of peer feedback in Chinese–English translation classes.

**Three levels of peer feedback**	**QTY (Quantity)**	**Percentage (%)**
Corrective feedback	377	73.63%
Processing-information	65	12.70%
Personal evaluation	70	13.67%
Total	512	100%

The results show that students gave almost equal amounts of feedback on personal evaluation and information processing, with the former scoring slightly better than the latter (0.97%). However, not all students gave general comments about personal evaluations or information processing. Nevertheless, they all mentioned at least five points for corrective feedback in the form of suggestions for change.

Moreover, 68 out of the 70 personal evaluations were purely laudatory and only two were directly critical. The results further imply that even under anonymity, affective factors still exert an influence. Students place great importance on interpersonal relationships and avoid negative comments.

This study of corrective feedback, the most commonly used strategy to reveal specific errors in translations of students, shows that direct and indirect feedback account for a predominant share of the total (more than 80%; see Table 2). Indirect corrective feedback is the most frequently adopted form (49.01%), followed by direct corrective feedback (38.99%), and finally, to a much lesser extent, metalinguistic corrective feedback, which accounts for only 49 cases (13.00%). Of the 49 metalinguistic feedback points, 47 took the form of a brief grammatical description. The use of an error code occurred only twice (see Table 1). The data show that the proportion of direct and indirect corrective feedback is more than 85% of the total, demonstrating that students prefer indirect and direct corrections and to a much lesser extent metalinguistic feedback. Indirect feedback is the most frequently used strategy: 9.02% more than direct feedback and 35.01% more than metalinguistic feedback.

**Table 2:** Types of peer corrective feedback in Chinese–English translation classes.

**Types of peer corrective feedback**	**QTY (Quantity)**	**Percentage (%)**
• Direct CF	148	38.99
• Indirect CF	180	49.01
• Metalinguistic CF	49	13.00
• Brief grammatical description	47	12.47
• Error code	2	0.53
**Focus of the feedback**		
• Focused CF	0	0.00
• Unfocused CF	377	100
• Electronic feedback	0	0.00
• Reformulation	0	0.00

All students in this study preferred an unfocused over a focused feedback strategy. There was no electronic feedback or reformulation.

Further investigation reveals that students provide direct and indirect feedback in different ways, rather than just correcting or pointing out errors. Students generally give direct feedback with an explanation. They give indirect feedback in a questioning way to show that they are unsure whether they are correct. Some also utilize two types of feedback: direct combined with indirect, direct combined with metalinguistic, or indirect combined with metalinguistic.

The following examples illustrate the strategies for corrective feedback that students adopted. Some direct feedback is shown as follows:

(a.) *summer-dog days; hat-hats*.
(b.) *Delete “stepping in.”*
(c.) “*a piece of” is literal, can be changed to “a sea of.”*
(d.) *It is better to modify “boasts in front of the friend” to “boasts to his friends.”*
(e.) “*old”—is not equal to the source text, “ancient” is better*.
(f.) *hot summer days-dog days (this is a fixed expression in English)*.
(g.) *Not just one “hat” but a sea of “hats.”*
(h.) “*is not dispersed” should use positive voice, “does not disperse.”*
(i.) “*gathered”—“gathers” inconsistent tenses*.
(j.) “*Mix” used as an adjective should be “mixed” instead of “mixing.”*

An examination of these examples of direct corrective feedback shows the tendency of students to immediately replace an incorrect word or phrase with the correct one like (a). They also provide direct feedback in the form of sentences, both in English and Chinese like (b and c). Students often employ direct feedback because it is the fastest and easiest way (Chandler, 2003). We also found that direct feedback usually targets superficial errors, such as spelling, wording, and punctuation. Students may find such errors so obvious that further explanation is unnecessary.

In some cases, students explained their corrections like (d, e, and f) in more detail. Sometimes, they combine direct feedback with metalinguistic feedback like (g, h, i, and j), giving a grammatical explanation. Some indirect feedbacks are listed as follows:

(a.) *In here*.
(b.) *was busy with (How can the stage be busy?)*.
(c.) *Does “young boy” mean “boy”?*
(d.) “*every social events,” grammar mistake*.
(e.) “*…conditions, in order to…” The use of commas or “in order to” is inappropriate*.
(f.) “*hit by these words” is not proper*.
(g.) *The translation “zichouyinmao” is not suitable here, and the annotation “(first four of the 12 Earthly Branches)” confuses readers*.
(h.) *The use of the word “ridiculous” is not suitable because it carries the meaning of “stupid.”*
(i.) *put subsistence right and development right of people at first (You would better use a more formal expression)*.
(j.) “*dry smoke,” as an alternative to “drought smoke,” is clearly nonsense Chinglish as well*.
(k.) “*Talking with you for one moment is much better than reading books for 10 years,” (It is over-literal). It should be adapted to be more like English*.
(l.) *At the beginning of the second paragraph, a word is ignored and missed translating*.

Indirect corrective feedback takes various forms, ranging from underlined errors like (a) to describe the errors using phrases like (b and d) or short sentences in English like (c, e, g, h, and i). Feedback showing pragmatic errors, such as Chinglish like (j), word-for-word translation like (k), or missing translations like (l) was also counted as indirect feedback. Apart from correcting language errors, feedback that focuses on the meaning of the text, such as pointing out the lack of translator of understanding of the text or violation of translation standards, is also indirect corrective feedback. Although, it is convenient to mark errors by underlining them or drawing a circle around them, only one student did so. The most common form of indirect feedback was the use of sentences or complete statements. Students often used full sentences such as “…is incorrect” or “…is not equal to the source text” when giving indirect feedback.

Regarding the case of direct feedback with explanations, there are also many cases of indirect feedback with additional notes, which involve comments, general suggestions, and personal opinions. By providing such specific explanations, the author can become aware of why these items were marked. Some metalinguistic feedbacks are illustrated as follows:

(a.) *Wrong fixed collocation: save your heart; as a going says*.
(b.) *I personally think the structure “not only…but also” is not proper here because, in the original text, the two things are simply side by side*.
(c.) *more fruits of development will be equally brought to all people, and their right to equal participation and development will be guaranteed (It is better to have the same subject in two sentences. And it may be easier for readers to understand if we translate it as “make sure that people can enjoy… People's rights and interests can be guaranteed…”)*.
(d.) *Noun phrases in the original text should not be translated as a sentence*.
(e.) *Violation of the elegance: The translated version lacks the necessary connection between sentences*.
(f.) “*poverty alleviation, safeguarding, and improving people's wellbeing, and developing all social programs.” Either all verb form or all noun form*.
(g.) “*and there are also boys like me who know nothing, the smell of drought smoke is not dispersed.” There is no logical connection between the two clauses. It is better not to translate in one sentence*.
(h.) “*…as the smell of perspiration and tobacco floating around the village lane” (There is no obvious relationship between the two clauses)*.

An observation of metalinguistic feedback examples reveals two primary forms of correction. There is a predominance of grammatical descriptions and only two error codes. Students write metalinguistic feedback more in Chinese than in English like (c, f, g, and h). When students give metalinguistic feedback, they use hedges much more often than in the case of direct and indirect feedback [e.g., “I personally think that… is better” like (b), or “maybe. a better choice”]. Some also directly expressed their uncertainty about a particular language point, such as “I don't know if it's right (or correct).” The limited language skills of students are most likely the cause of their tentative language use. It may also indicate the self-doubt of students when they disagree with their peers about their understanding of the source texts. However, there is still metalinguistic feedback that clarifies the nature of errors of their peers like (a, d, and f).

### Influence of Peer Feedback on the Revised Translations

The data revealed that peer corrective feedback had a positive impact on the translations of students. All students made at least one correct revision following the comments. In contrast, peer feedback at the first and second levels was not effective, as no revisions were made in response to these two types of feedback. Of the 377 instances of corrective feedback, 252 resulted in a change, 66.84% of the total (see Table 3). The strategy of direct corrective feedback seemed to have the highest adoption rate, with 132 (89.19%) items revised. However, such a high rate may mean that students did not seek out more information about their mistakes. They adopted the advice of their peers out of laziness and without confirming its correctness.

**Table 3:** Responses and revisions of students.

**Revisions to different types of CF**	**QTY (Quantity)**	**The adoption rate (%)**
Revisions to direct feedback	132	89.19%
Revisions to indirect feedback	95	52.78%
**Revisions to**		
Brief grammatical description	25	51.02%
Total	252	66.84%

Metalinguistic feedback was the least used correction strategy. Only 25 items (51.02%) of the short grammatical description were modified, while none of the two error codes led to final revisions. The proportion of indirect corrective feedback applied was also insignificant: only 95 (52.78%). It is likely that students who received indirect or metalinguistic corrective feedback still did not know how to correct their errors, even though they realized that the marked places needed improvement. This may be due to some students that were uncertain about the feedback because they had not received clear and standard answers. In the end, they insisted that their version was better than their peers. The students did not seem inclined to study these indirect corrections more closely. Consequently, they simply skipped them.

We analyzed the correctness of revisions of students using different types of peer corrective feedback. Connor and Asenavage (1994) claimed that in terms of the number of revisions, the validity of peer feedback is not significant compared with teacher feedback and self-correction. However, we found students could significantly benefit from peer corrective feedback if they made appropriate changes according to the suggestions of their peers. As shown in Table 4, of the total 252 corrections, 217 (86.11%) corrections following peer corrective feedback were successful. But there were also 35 (13.89%) items that were not successful, with 17 (6.75%) cases of no-change and 18 (7.14%) regressions.

**Table 4:** Correctness of revisions of students into different types of peer corrective feedback.

**Evaluation of revisions**	**Revisions (252 in total)**	**Revisions to direct CF**	**Revisions to indirect CF**	**Revisions to metalinguistic CF**
Improved	217 (86.11%)	116 (87.88%)	82 (86.32%)	19 (76%)
Regressed	18 (7.14%)	8 (6.06%)	8 (8.42%)	2 (8%)
No Difference	17 (6.75%)	8 (6.06%)	5 (5.26%)	4 (16%)

Further examination of the data suggests that the most useful peer feedback is direct and indirect corrective peer feedback. Of the 132 cases, 116 (87.88%) students improved. Similarly, 86.32% of revisions to indirect corrective items resulted in improvements. Interestingly, although direct feedback provided students with explicit corrections, 16 corrections (12.12%) were wrong. Some of these were due to incorrect feedback from fellow students due to their limited language skills.

In contrast, only 76% of the adjustments made according to a short grammar description were correct. This shows that metalinguistic feedback is not only a challenge for students to give but also to benefit from it in the right way. When receiving such feedback, students may not understand it or may not yet know what is correct.

We not only assessed the correctness of each revision but also graded the first and last translations of students from a comprehensive perspective according to the scoring standard for translations in CET-4 and CET-6. This scoring standard evaluates translations mainly based on fidelity, fluency, sentence structure, word usage, and the number of grammatical errors. Our study showed that students made superficial revisions that reduced grammatical errors and incorrect expressions, as well as text-based revisions that reflected greater similarity to the source text and improved fluency and coherence. Most students received higher marks for their final translations than for their first drafts. This finding further demonstrates that peer feedback has a positive impact on translation quality.

### The Influence of Text Genres on Peer Feedback

We also found that there were differences in the extent of peer feedback (see Table 5). The story in ancient Chinese led to more corrective feedback (42.48%) than the other two assignments. The excerpt from a government report received less feedback (23.28%). The assignment in ancient Chinese was the least familiar to the students, so they had to make an effort to understand the meaning of the story before starting the translation. Subjective understanding and different interpretations led to different views.

**Table 5:** Peer corrective feedback from different text genres.

**Text genres**	**Direct CF**	**Indirect CF**	**Metalinguistic CF**	**Total**	**Percentage**
Ancient Chinese story	51	92	17	160	42.48%
Chinese literature	52	54	23	129	34.24%
Government report	45	34	9	88	23.28%

As a result, students expressed different opinions and gave more indirect and metalinguistic feedback (68.13%) on this text. The language in the government work report was much simpler and more direct. Also, the content was much more familiar. Moreover, fixed translations of the keywords in this text were available on the Internet. Of the first 30 drafts, most of the translations of students were essentially the same and differed only in minor ways. Therefore, it was not surprising that this section underwent the fewest corrections.

## Discussion and Conclusion

### Discussion

In our quasi-experiment, we studied peer feedback systematically. Although students made mistakes, they provided both surface- and text-based feedback. Moreover, to make their feedback clearer, students used mixed corrective strategies. They expressed their own ideas when they marked an error and added a brief grammatical description or explanation. They wrote corrections in complete sentences, gave individual interpretations, and showed a strong sense of solidarity. Students gave more feedback, especially text-based, on the second assignment and less on the third. Such a discrepancy implies that text genres can influence peer feedback. Texts that require a deeper understanding and a subjective interpretation elicit more feedback than texts that are factual and close to the living environment of students.

With computer assistance, there is no invalid feedback due to poor handwriting and illegible mark. However, most personal evaluations are pure praise and few are critical suggestions, which may not result in the improvement of students in the long run. This finding implying affective considerations can only be reduced rather than eliminated, even under online anonymity.

Regarding the influence of peer feedback on the translations, this study found that peer feedback improved the overall quality, which echoed the study by Yu et al. (2020), although the latter is a study on English–Chinese translation. Students not only did reduce grammatical errors but also made textual revisions based on feedback that involved the ideas and opinions of others. Most students earned higher grades for their revised papers. This shows that peer feedback activity is an effective way of collaborative learning.

Another point worth noting is that students who received lower grades in their first versions made more revisions and achieved significant progress in their final versions. This finding is consistent with what Wu (2019) concluded from previous studies: students with less language proficiency do not have sufficient knowledge to recognize or correct language problems, making them more likely to be mere feedback recipients. As mentioned, Allen and Mills (2014) also found that reviewers with better language skills offer more feedback. Thus, we may conclude that in advanced translation classes, the language proficiency of students plays a significant role in peer feedback.

Finally, there are differences in the extent of peer feedback with regard to translation text genres. Texts that demand deep comprehension and subjective interpretation lead to radically different translation versions. Consequently, the corresponding translated texts receive much more feedback, especially indirect and metalinguistic feedback. Students may also use hedges (i.e., “I think” and “Maybe”) to demonstrate their own opinions. On the contrary, there is minimal room for subjectivity in those official and rigorous texts, which contain many fixed expressions and technical terms. The translation of students is largely identical and only has minor differences. As a result, it is not surprising that feedback from this kind of texts is generally at surface level and the number of corrections is much fewer.

It is important to note that only corrective peer feedback had a positive impact on the texts of students. Peer feedback of both personal evaluation and information processing was not effective, as students did not make any changes. This finding contradicts Hattie and Timperley (2007), where they argued that feedback with processing information is the most conducive to student revision and that too much corrective feedback does not achieve the desired effect. However, our findings are consistent with their view that mere praise does not improve learning outcomes.

Of all the corrective feedback given, the quasi-experiment showed that direct corrective feedback was the most helpful to students, with 132 (89.19%) points taken up and 116 (87.88%) points improved. These data are consistent with the findings of Chandler (2003) that direct feedback is the most effective. The high adoption rate of direct feedback may be due to the minimal processing requirements (Ellis, 2009:148) and the fact that it is easier for students to revise.

Students responded less well to indirect and metalinguistic corrective feedback. This kind of feedback requires students to “reflect about linguistic forms” and “engage in deeper processing” (Ellis, 2009:146). Therefore, students may be reluctant to engage more deeply with those meandering instructions. When receiving indirect or metalinguistic corrective feedback, students may remain ignorant of how to correct their texts (Zhang et al., 2019). Only 76% of revisions through short grammatical descriptions were successful. This suggests that metalinguistic feedback is not only a challenge for translation teachers (Ellis, 2009) but also difficult for others to make full use of it. Indirect and metalinguistic feedback involves much more hedging than direct corrections, and these can be more difficult for learners to interpret (Baker and Bricker, 2010). In the absence of clear standard answers, learners are skeptical of this kind of feedback and believe that their translations do not need to be revised.

Despite the improvement in corrections, there were still some unsuccessful revisions. Most of the ineffective feedback was due to the anxiety generated by peer feedback. As Dörnyei (1994) stated, L2 learners with intrinsic motivation study language to pursue the pleasure of learning and to satisfy their curiosity. In contrast, learners with extrinsic motivation are interested in higher scores and rewards and want to avoid punishment. He also claimed that intrinsic motivation could facilitate second language learning. In addition to motivation, anxiety also plays a crucial role. According to Jain and Sidhu (2013), we can divide anxiety into facilitative and debilitating anxiety. Facilitative anxiety motivates learners to make an effort to learn a language, while debilitating anxiety causes them to avoid the language learning process (Zhang, 2001). Students did not seem intrinsically motivated by peer feedback but still wanted to get good grades. After they were told that their feedback on the corrections of other people and revisions would count toward the overall score, they gained extrinsic motivation and facilitative anxiety to give feedback and make revisions. However, the large number of ambiguous and incorrect corrections suggests that students were not sufficiently motivated to seek rigorous corrections that addressed issues beyond their current knowledge. Some passive learners often blindly adopted the suggestions of others to demonstrate that they had revised the feedback in their final text. Some even followed, without any reflection, clearly incorrect instructions.

### Conclusion

This quasi-experiment points out several implications for translation training by examining what types of peer feedback are useful in an advanced Chinese–English translation class with computer assistance and how this affects translation quality. Since imparting knowledge is only one aspect of teaching, teachers should also consider the sociocultural aspects of teaching. This requires teachers to create opportunities for students to co-construct and understand knowledge by engaging in social interaction. Tefera et al. (2020) suggest that teachers should focus on teaching methods, such as collaborative learning, that improve academic performance and learning satisfaction of university students. Previous empirical studies in English as a Foreign Language (EFL) contexts also claim that Chinese education is ready to replace traditional teacher-centered classrooms with more student-centered teaching methods (Zhang, 2019).

Peer feedback is a typical activity that can fulfill this task. Since the findings suggest that peer feedback has a positive impact on the quality of translations of students, it should be organized regularly. It would ease the correction burden of the teacher. However, as with other collaborative learning activities, peer feedback requires prior training. Teachers can teach students different feedback strategies. As Ellis (2009:215) states, direct feedback is useful when students revise their work, but it is unlikely to benefit students in the long run. Indirect feedback is a kind of guided learning and problem-solving practice. It is therefore conducive to long-term student learning, as it requires in-depth information processing. For students to benefit, teachers should encourage them to provide both direct and indirect corrective feedback. Although students in this study used only about half of the indirect corrective feedback, the revision accuracy rate was 86.32%. The results of this study suggest that students may benefit more from indirect peer corrective feedback if they need to process it carefully. Moreover, electronic devices should be utilized in peer feedback so to facilitate the efficiency of reviewing and to reduce illegible marks. To further motivate students and create facilitative anxiety, instructors could stipulate that the quality of their feedback and revisions make up a certain percentage of their final grades in order to avoid misleading and ineffective feedback as well as ineffective corrections.

### Limitations and Implications

The limitations to this study are as follows: first, we only collected data from 30 participants. Second, the study was only with English majors as subjects. It cannot be generalized to a wider range of students. Third, external factors, such as personality, gender, perception of peer feedback, and student language proficiency, can influence the feedback of students. Fourth, although there are suggested answers for the three translation tasks, they do not dictate fixed criteria. Since translations are flexible, other versions may also be acceptable. Subjective factors are inevitable in assessing the quality of translations of students. Finally, in L2 teaching, the timing of feedback also plays a role. Immediate and delayed corrective feedback helps language learners to different degrees. However, this study focused only on delayed corrective feedback and did not address the differences between immediate and delayed corrective feedback.

Future studies may recruit a larger sample in response to these limitations, involve participants from different fields of study or colleges, or conduct experiments in elementary and secondary schools. We also suggested that other classroom case studies consider establishing a control group to validate the results of this experiment. A variety of external factors can influence peer feedback. Immediate corrective feedback and delayed corrective feedback have different levels of impact. To gain a deeper understanding of peer feedback, it is necessary to further investigate these mediating factors.

## Data Availability Statement

The original contributions presented in the study are included in the article/supplementary material, further inquiries can be directed to the corresponding author/s.

## Ethics Statement

The studies involving human participants were reviewed and approved by School of Foreign Language Studies, Chang'an University. The patients/participants provided their written informed consent to participate in this study.

## Author Contributions

ZL and XS designed and directed the project, and wrote the draft. XS, JG, ZL, and FW did the data collection and data analysis. All authors discussed the results and contributed to the final manuscript.

## Conflict of Interest

The authors declare that the research was conducted in the absence of any commercial or financial relationships that could be construed as a potential conflict of interest.

## Publisher's Note

All claims expressed in this article are solely those of the authors and do not necessarily represent those of their affiliated organizations, or those of the publisher, the editors and the reviewers. Any product that may be evaluated in this article, or claim that may be made by its manufacturer, is not guaranteed or endorsed by the publisher.
